# Long-Term Outcomes After Guillain-Barré Syndrome: A Systematic Review of Functional Recovery, Residual Symptoms, and Quality of Life

**DOI:** 10.7759/cureus.106509

**Published:** 2026-04-06

**Authors:** Sharafath Hussain Zahir Hussain, Zaw Moe

**Affiliations:** 1 Internal Medicine, Milton Keynes University Hospital NHS Foundation Trust, Milton Keynes, GBR; 2 Neurology, Milton Keynes University Hospital NHS Foundation Trust, Milton Keynes, GBR

**Keywords:** all neurology, guillain-barré syndrome, narrative review, quality of life, review article, systematic review

## Abstract

Guillain-Barré syndrome (GBS) is an acute immune-mediated polyradiculoneuropathy characterized by rapidly progressive weakness and variable recovery trajectories. Although many patients regain functional independence after the acute phase, increasing evidence suggests that long-term sequelae such as fatigue, pain, residual disability, and reduced health-related quality of life are common and frequently under-recognized. However, contemporary evidence on long-term outcomes after GBS remains dispersed across multiple study designs and populations, making an updated synthesis clinically relevant. This systematic review aimed to evaluate long-term outcomes reported at or beyond six months after GBS onset, focusing on functional recovery, persistent symptoms, and patient-reported quality of life. A systematic search of PubMed was conducted for studies published between January 2014 and November 2025 using predefined search terms related to GBS and long-term outcomes. Studies reporting outcomes beyond six months after disease onset were included, encompassing cohort studies, clinical trials with follow-up, qualitative studies, and systematic reviews. Due to heterogeneity in study design, outcome measures, and follow-up duration, findings were synthesized narratively. Across the included literature, most patients demonstrated substantial neurological recovery within the first year; however, a clinically meaningful proportion continued to experience persistent symptoms beyond one to three years. Fatigue and neuropathic pain were among the most frequently reported long-term sequelae and were major contributors to impaired quality of life despite apparent motor recovery. Residual functional limitations, participation restrictions, and psychosocial impacts were also commonly reported. Factors associated with poorer long-term outcomes included older age, severe disease at presentation, requirement for mechanical ventilation, autonomic dysfunction, and axonal electrophysiological patterns. These findings highlight that recovery from GBS is often incomplete despite improvement in neurological function. Long-term follow-up incorporating rehabilitation, symptom-directed management, and patient-reported outcome measures may be essential to better address persistent morbidity and optimize quality of life in GBS survivors.

## Introduction and background

Guillain-Barré syndrome (GBS) is a rapidly progressive, immune-mediated polyradiculoneuropathy that frequently follows an antecedent infection and can result in respiratory failure requiring intensive care [1]. Standard acute treatments, including intravenous immunoglobulin and plasma exchange, reduce disease severity and accelerate recovery in appropriately selected patients; nevertheless, recovery remains heterogeneous, and many survivors report ongoing symptoms long after hospital discharge [1-4].

GBS comprises several clinical subtypes, including acute inflammatory demyelinating polyradiculoneuropathy (AIDP), the most common form, as well as axonal variants such as acute motor axonal neuropathy (AMAN) and acute motor-sensory axonal neuropathy (AMSAN), which differ in electrophysiological features and geographic prevalence [1]. Historically, GBS has been regarded as a monophasic disorder with a generally favorable prognosis. However, emerging literature demonstrates that functional independence does not necessarily equate to full recovery. Persistent fatigue, pain, sensory symptoms, reduced endurance, limitations in daily activities, and psychological consequences may persist for years after apparent neurological improvement [2,3]. Furthermore, commonly used disability scales may inadequately capture participation restrictions and patient-perceived recovery [4].

Large prospective initiatives such as the International Guillain-Barré Syndrome Outcome Study were developed to standardize outcome measurement and identify predictors of disease course and long-term recovery, including patient-reported outcomes [5]. Over the past decade, multiple cohort studies, systematic reviews, and qualitative investigations have expanded understanding of long-term functional status, symptom burden, and health-related quality of life across diverse populations [6]. Rehabilitation-focused studies further highlight that despite functional gains, fatigue and neuropathic pain frequently persist and remain key drivers of long-term impairment [6-9].

## Review

Methodology

This systematic review with narrative synthesis was conducted in accordance with the Preferred Reporting Items for Systematic reviews and Meta-Analyses (PRISMA) 2020 guidelines [10]. A systematic PubMed search was performed for studies published between January 1, 2014, and November 30, 2025. The PubMed search strategy used combinations of keywords and Boolean operators as follows: (“Guillain-Barre syndrome” OR “Guillain-Barré syndrome” OR GBS) AND (“long-term outcome” OR “long term outcome” OR follow-up OR recovery OR fatigue OR pain OR “quality of life” OR “patient-reported outcome” OR “patient reported outcome”). Reference lists of relevant included articles were also screened to identify additional eligible studies. Title and abstract screening, full-text eligibility assessment, and data extraction were performed by the two authors, with disagreements resolved through discussion and consensus. The literature search was limited to the PubMed database. A formal risk-of-bias assessment was not performed because this review synthesized a heterogeneous body of evidence that included cohort studies, reviews, qualitative studies, and a protocol, limiting the applicability of a single standardized appraisal tool across all included article types. This review was not prospectively registered.

Eligibility Criteria

Eligible studies were PubMed-indexed and involved adult or pediatric GBS cohorts, clinical trials with follow-up, qualitative studies of long-term patient experience, or systematic reviews reporting outcomes at or beyond six months after GBS onset, with preference for follow-up of at least 12 months when available. Case reports and very small case series without generalizable long-term outcome data were excluded, as were studies focused solely on acute outcomes within six months and studies in which GBS outcomes could not be separated from other neuropathies unless subgroup-specific outcomes were clearly reported. Given the heterogeneity and relative scarcity of long-term outcome literature in GBS, diverse article types relevant to long-term recovery, symptom burden, and patient-reported outcomes were included for qualitative narrative synthesis. Original cohort and observational studies were prioritized, while selected systematic reviews were included where they provided complementary synthesis on long-term outcomes or patient-reported measures not fully captured by individual primary studies.

Outcomes and Data Extraction

Primary outcome domains were defined a priori and included functional recovery and disability (for example, Hughes functional grading), persistent symptoms (particularly fatigue and pain), and health-related quality of life and patient-reported outcomes (for example, 36-Item Short Form Health Survey, EuroQol 5-Dimension questionnaire, fatigue and pain-related instruments). Secondary considerations included prognostic factors associated with poor long-term disability or reduced quality of life, as well as the impact of rehabilitation and the persistence of symptoms despite physical recovery.

**Figure 1:**
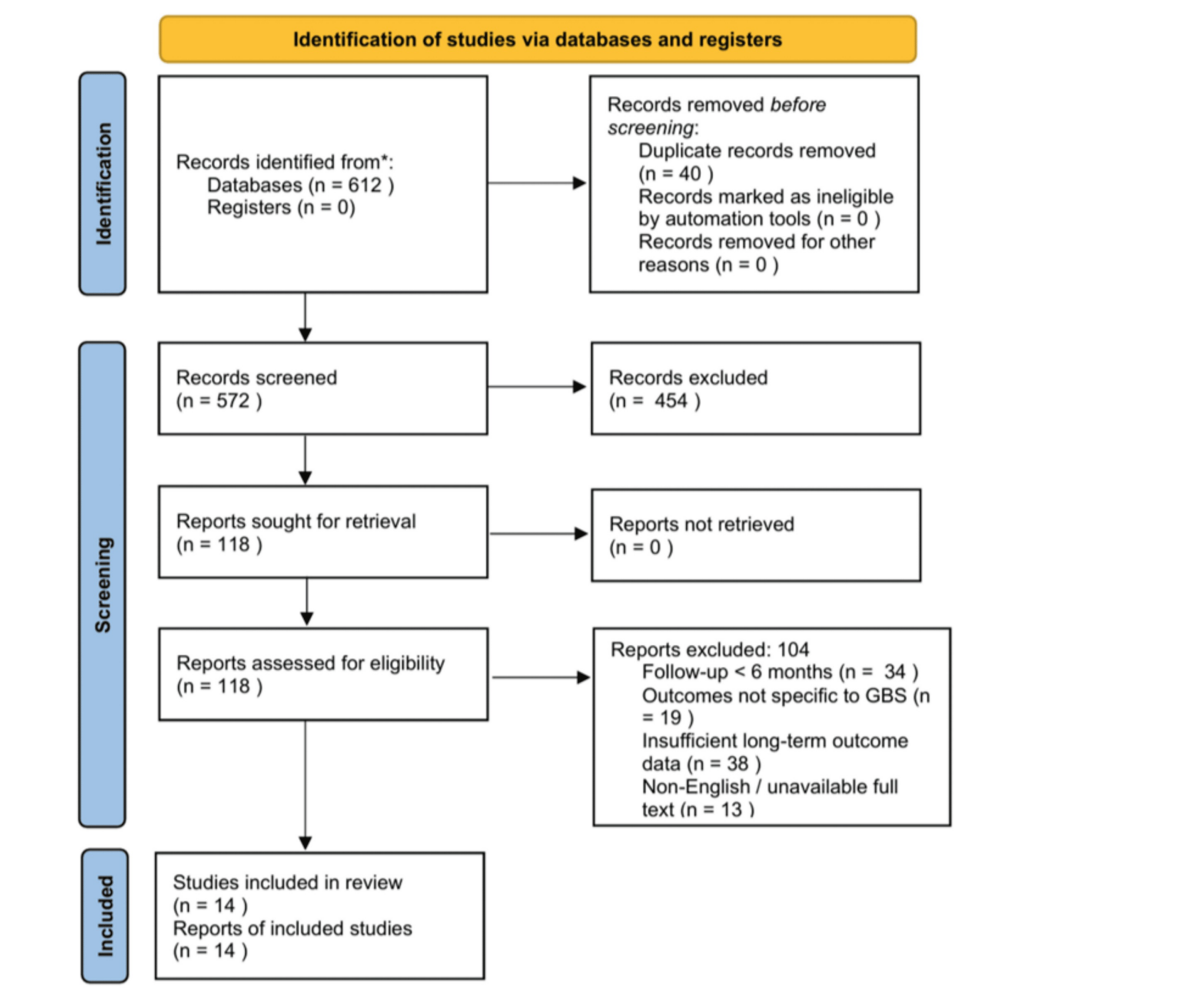
Preferred Reporting Items for Systematic reviews and Meta-Analyses (PRISMA) 2020 flow diagram of the study selection process. Flow diagram illustrating the identification, screening, eligibility assessment, and inclusion of studies in this systematic review according to PRISMA 2020 guidelines. Records were identified through PubMed database searching and citation screening, followed by duplicate removal, title and abstract screening, full-text eligibility assessment, and final inclusion of studies for qualitative synthesis.

For each eligible study, key variables were extracted, including study design, population, sample size, follow-up interval, outcome measures, and main long-term findings. Due to heterogeneity in study design, outcome measures, and follow-up duration, a narrative synthesis was performed rather than a meta-analysis. For narrative synthesis, findings were grouped according to predefined outcome domains, including functional recovery and disability, persistent symptoms such as fatigue and pain, health-related quality of life and patient-reported outcomes, prognostic factors, and rehabilitation- or participation-related outcomes. Conclusions were derived by identifying recurrent patterns and areas of convergence across the included studies within each domain. The study selection process is summarized in Figure 1.

Results

A total of 14 studies met the inclusion criteria and were included in the qualitative narrative synthesis. These comprised prospective and retrospective cohort studies, systematic and narrative reviews, a prospective cohort protocol, and qualitative studies examining long-term outcomes following GBS. Of the 14 included studies, eight were primary studies with participant-level data, collectively representing 1,198 participants, while six were secondary sources or study protocols. The relatively small number of included studies reflects both the limited availability of literature specifically addressing long-term outcomes after GBS and the application of predefined eligibility criteria focused on studies reporting outcomes at or beyond six months. The included studies evaluated functional recovery, persistent symptoms such as fatigue and pain, and health-related quality of life in diverse patient populations. Key characteristics and findings of the included studies are summarized in Table 1.

**Table 1:** Summary of included studies evaluating long-term outcomes after GBS. GBS = Guillain-Barré syndrome; CIDP = chronic inflammatory demyelinating polyneuropathy; PROM = patient-reported outcome measure; HRQoL = health-related quality of life; FIM = Functional Independence Measure; 6MWT = six-minute walk test; mRS = modified Rankin Scale; ICF = International Classification of Functioning; SF-36 = 36-Item Short Form Health Survey; EQ-5D = EuroQol 5-Dimension questionnaire

Study (first author, year)	Design	Population	Follow-up	Key outcomes measured	Main long-term findings
Willison et al., 2016 [1]	Narrative review	Global GBS literature	N/A	–	Overview of GBS pathophysiology, treatment, and prognosis, highlighting that approximately 20% of patients may have persistent disability despite treatment
Bellanti and Rinaldi, 2024 [2]	Comprehensive review	Global GBS population	N/A	–	Describes mechanisms and clinical features of GBS and emphasizes residual deficits and reduced quality of life in a subset of survivors
Merkies and Kieseier, 2016 [3]	Narrative review	GBS and CIDP	N/A	Fatigue, pain, anxiety, depression	Fatigue and pain are common and may persist for years, significantly impacting quality of life
Pelouto et al., 2025 [4]	Systematic review	Polyneuropathy patients including GBS	N/A	PROMs for HRQoL	Identified multiple PROM instruments but highlighted limited validation for GBS-specific outcomes
Jacobs et al., 2017 [5]	Prospective cohort protocol	International GBS Outcome Study (IGOS)	≥1 year planned	Disability scales and prognostic indicators	Describes large international cohort designed to identify predictors of disease course and long-term outcomes
Uz et al., 2023 [6]	Prospective cohort	24 adult GBS patients in rehabilitation	3 years	Hughes scale, FIM, 6MWT, fatigue scale	Significant functional improvement but persistent fatigue and pain remained common
Acero-Garcés et al., 2024 [7]	Retrospective cohort	45 post-Zika GBS patients	Median 28 months	mRS, Barthel Index, disability scales	Majority regained independent ambulation, though some residual deficits remained
Walteros et al., 2019 [8]	Prospective cohort	34 Zika-associated GBS patients	Median 17 months	Hughes scale, HRQoL surveys	Most patients recovered motor function but had higher rates of depression and disability than controls
Berisavac et al., 2020 [9]	Multicenter cohort	74 GBS patients	6 months	SF-36 and disability scales	Disability improved over time but quality of life recovery lagged behind physical improvement
Darweesh et al., 2014 [11]	Systematic review	14 long-term QoL studies	N/A	HRQoL domains	Many patients experienced persistent physical limitations and reduced quality of life
Forsberg et al., 2015 [12]	Qualitative study	35 GBS survivors	~2 years	Patient-reported recovery experiences	Identified themes of adaptation and ongoing functional limitations
Rath et al., 2021 [13]	Retrospective cohort	121 GBS patients	~1 year	Disability change and treatment exposure	Residual disability persisted in a proportion of patients despite treatment
Verboon et al., 2021 [14]	Observational cohort	221 mild GBS patients	1 year	Walking ability and residual symptoms	Many patients with mild GBS reported ongoing symptoms despite functional recovery
Papri et al., 2024 [15]	Prospective cohort	644 GBS patients (Bangladesh)	26 weeks (6 months)	Pain (numeric rating scale), disease severity, HRQoL (EQ-5D)	Pain was common at enrolment and persisted in a meaningful proportion at 13 and 26 weeks; pain severity correlated with disease severity and was associated with worse HRQoL domains (self-care, usual activities, anxiety/depression)

Discussion

Functional Recovery and Disability Trajectories

Epidemiological studies indicate that approximately 20% of patients experience persistent disability following GBS, even after standard treatment with intravenous immunoglobulin or plasma exchange [1]. Across cohorts, most patients demonstrate the greatest recovery within the first six to 12 months, but a clinically important minority experience residual disability beyond this period [6,7,9,11,12]. In a three-year follow-up study of adults after inpatient rehabilitation, functional scores improved between discharge and one-year and three-year follow-up assessments, yet persistent symptoms and incomplete recovery remained evident for some patients [6]. Cohorts following Zika-associated GBS similarly document improvement in ambulation and disability scores over time, but also identify residual activity limitations and participation restrictions [7,8].

Evidence also suggests that “mild” impairment on global disability scales can coexist with meaningful symptom burden. Patients who regain independent ambulation may still report limitations in endurance, higher-level mobility, or fine motor activities, indicating that neurological “recovery” may be incomplete when assessed from the patient perspective [6,9,12,13]. Real-world observational data further underscore heterogeneity in outcomes over time, with severe cases tending toward slower or less complete long-term recovery [13].

Persistent Fatigue, Pain, and Symptom Burden

Fatigue and pain are repeatedly identified as dominant long-term sequelae. A focused review addressing fatigue, pain, anxiety, and depression in inflammatory neuropathies emphasizes that fatigue and pain can persist for years following GBS, significantly affecting well-being even when routine neurological examination appears reassuring [3]. In rehabilitation and cohort studies, persistent fatigue and neuropathic pain remain prevalent at long-term follow-up and can meaningfully impair endurance and participation despite gains in functional independence [6-8]. This symptom burden highlights a key limitation of follow-up focused solely on strength or basic gait endpoints, because persistent fatigue and pain may represent ongoing morbidity even among individuals categorized as functionally “independent” [3,6,9,11].

Health-Related Quality of Life and Patient-Reported Outcomes

Health-related quality of life outcomes after GBS are influenced by physical limitations, fatigue, pain, mood symptoms, and participation restrictions [3,6,9,11]. Longitudinal evidence suggests that improvements in objective disability scores do not always translate into proportional improvements in health-related quality of life, and quality-of-life recovery may lag behind motor recovery [6,9]. This is a central clinical finding of the review and highlights the importance of assessing recovery not only through disability scales but also through persistent symptoms, participation, and patient-reported outcomes. Qualitative evidence adds important insight into these patient-centered outcomes, emphasizing ongoing adaptation to bodily restrictions, altered self-identity, and the long-term work of “balancing” everyday life after illness [12].

Patient-reported outcome measure-focused research also highlights measurement challenges. A systematic review of Patient-reported outcome measures used in GBS and related inflammatory neuropathies identified multiple instruments but found persistent limitations in content validity across available tools, supporting the need to improve measurement strategies for long-term follow-up and patient-centered care [4].

Prognostic Factors for Poorer Long-Term Outcomes

Across the broader GBS literature, predictors associated with poorer long-term outcomes include older age, higher disease severity at nadir, requirement for intensive care or mechanical ventilation, autonomic dysfunction, and electrophysiological features consistent with axonal injury [1,5]. Standardized initiatives such as the International Guillain-Barré Syndrome Outcome Study aim to refine prognostic accuracy by linking early clinical characteristics to later disability and patient-reported outcomes [5]. Contextual factors may also influence outcomes; for example, limited access to rehabilitation services and socioeconomic constraints have been associated with worse long-term functional or participation outcomes in some cohorts [7].

Evidence on treatment exposure and long-term outcomes suggests persistent heterogeneity, including in severe disease, in which multiple acute treatment courses did not clearly translate into improved long-term motor outcomes in real-world practice [13]. Conversely, among mild GBS cases, observational data suggest that differences in some long-term endpoints between those treated and untreated may be limited, while residual symptoms can still occur even in mild disease [14]. Additionally, prospective cohort data indicate that pain remains an important determinant of reduced quality of life after GBS, underscoring that long-term burden may persist even when motor recovery is otherwise favorable [15].

Pathophysiological Mechanisms Underlying Persistent Symptoms

While the studies included in this review describe the frequency and clinical pattern of long-term disability, fatigue, pain, and reduced quality of life after GBS, understanding the biological basis of these persistent sequelae is also important. A mechanistic perspective helps explain why some patients continue to experience substantial symptom burden despite apparent motor recovery on conventional disability scales and provides context for the heterogeneity observed in long-term outcomes.

GBS is typically triggered by a preceding infection that initiates an aberrant autoimmune response [16-25]. Molecular mimicry between microbial antigens and peripheral nerve components leads to activation of complement pathways and recruitment of macrophages and lymphocytes [17,19,20,22,24]. In the classic AIDP variant, immune cells attack Schwann cell membranes, causing segmental demyelination of peripheral nerves [17-19,22]. This demyelination slows saltatory conduction and may progress to conduction block; macrophage-mediated stripping of myelin is a hallmark of this process [17-19,22]. Axonal variants such as AMAN involve direct antibody-mediated attack on axolemmal gangliosides; complement fixation leads to membrane attack-complex formation and Wallerian degeneration of motor axons [17,19-22,24].

Although remyelination and axonal regeneration occur during recovery, they are often incomplete [17-22]. Remyelinated segments have thinner myelin sheaths and shorter internodes, resulting in chronically reduced conduction velocity and early fatigability [17-19,21,22]. Slow axonal regeneration in AMAN can leave residual denervation and contribute to weakness and reduced endurance years after the acute illness [17,19-22]. Persistent immune activation, cytokine release, and dysregulation of autonomic function may further impair neuromuscular transmission and contribute to chronic fatigue and pain [16,17,20,22-24]. Central mechanisms, including altered central motor drive and maladaptive cortical plasticity, are postulated to play a role in persistent fatigue and reduced quality of life [16,17,20,23].

Understanding these mechanisms helps clinicians interpret long-term outcome studies. Apparent functional recovery on disability scales may coexist with ongoing physiological impairment [16,22,23]. Patients with significant axonal degeneration may achieve independent ambulation yet experience early fatigue during sustained activity, neuropathic pain, and reduced exercise tolerance [16,21-24]. Recognizing the biological basis of these sequelae supports a holistic approach to rehabilitation and informs future research aimed at neuroprotective or immunomodulatory strategies [16,17,22-24].

Rehabilitation, Participation, and Long-Term Functional Recovery

Emerging literature also highlights the importance of real-world participation and community-based outcomes in shaping long-term recovery after GBS. A national cross-sectional survey from the United Kingdom identified falls as an important issue among community-dwelling adults after GBS, underscoring that apparent motor recovery does not necessarily equate to safe or confident mobility in daily life [26]. Earlier natural history data similarly demonstrated substantial heterogeneity in recovery and identified clinical severity as an important determinant of prognosis [27]. Additional literature has also questioned whether long-term prognosis is determined solely by acute treatment exposure, suggesting that recovery trajectories are likely influenced by a broader interaction between disease severity, residual symptoms, rehabilitation access, and psychosocial adaptation [28]. A systematic review of exercise-based interventions further suggests that rehabilitation may improve functional performance, mobility, and endurance, although the available evidence remains heterogeneous and limited by small study sizes and variation in intervention design [29]. Patient-centered literature also emphasizes that recovery extends beyond neurological examination and disability grading, with a recent scoping review describing the biopsychosocial impact of GBS on independence, social participation, emotional well-being, and self-identity [30].

Rehabilitation-focused reports provide additional support for this broader understanding of recovery. Case-based evidence suggests that interventions such as robotic gait-assisted training may improve physical fitness, mobility, body composition, and quality of life in selected patients, although such findings should be interpreted cautiously given their limited generalizability [31]. Broader prognostic literature also reinforces the clinical heterogeneity of GBS and supports the need for individualized long-term follow-up based on disease severity and other risk factors [32]. More recent prospective observational work has continued to identify variability in presentation and outcome, with prognostic implications linked to disease severity and associated clinical features [33]. Similarly, structured rehabilitation programs have been reported to support functional recovery in individual cases, again highlighting the potential role of multidisciplinary rehabilitation in optimizing longer-term outcomes [34]. Cohort data from other settings also support the broader observation that GBS remains clinically heterogeneous, with outcomes varying across populations and care environments [35]. Taken together, these findings support a broader approach to long-term outcome assessment that incorporates participation, falls risk, rehabilitation needs, and patient experience alongside traditional disability measures.

Limitations

This review has limitations. Outcome measures, follow-up intervals, and study designs varied substantially across included articles, and the evidence base was methodologically heterogeneous, incorporating cohort studies, reviews, qualitative work, and a protocol. The included studies also differed in population characteristics, disease severity, clinical context, follow-up duration, and the outcome measures used, which limits direct comparability across studies and reduces the strength and generalizability of cross-study conclusions. Many articles were not primarily designed to capture long-term patient-centered outcomes. In addition, the search was limited to the PubMed database and to English-language accessible literature, which may have excluded relevant studies indexed elsewhere. Because of heterogeneity in populations, outcome measures, and follow-up duration, formal meta-analysis was not performed. A formal risk-of-bias assessment was also not performed, as the included evidence base was methodologically heterogeneous, limiting the applicability of a single standardized appraisal tool across all article types. Nevertheless, convergence of evidence across cohort, review, patient-reported outcome measures, and qualitative data supports the central finding that long-term symptom burden and quality-of-life impairment are not rare and may be underestimated by conventional disability scales [3,4,6-9,11].

From a clinical perspective, long-term follow-up should extend beyond strength and basic ambulation endpoints and incorporate systematic assessment of fatigue, pain, and patient-reported outcomes to identify unmet needs and target rehabilitation and symptom management strategies [3,4,6,9].

## Conclusions

Long-term outcomes after GBS are heterogeneous and extend beyond motor recovery alone. Although many patients regain functional independence, persistent fatigue, pain, reduced quality of life, and participation restrictions remain common. These findings highlight that improvement in objective disability does not necessarily equate to full patient-perceived recovery. Long-term follow-up should therefore incorporate patient-reported outcomes and rehabilitation-focused assessment in addition to conventional disability scales. Future research should prioritize longitudinal studies using standardized and validated outcome measures to improve comparability across studies and strengthen patient-centered prognostication after GBS.
